# Recurrent parasitemias with artemisinin partial resistance mutations during the 2024 Ethiopia malaria resurgence: a case series

**DOI:** 10.21203/rs.3.rs-9094035/v1

**Published:** 2026-03-18

**Authors:** Dessalegn Geleta, Bokretsion G. Brhane, Adugna Abera, Mahlet Belachew, Heven Sime, Atsbeha Gebreegziaxher, Melak Getu, Abraham Ali, Neamin Tesfaye, Medhanye Habtetsion, Belayneh Kokobe, Mandefro Kebede, Zemene Worku, Geremew Tasew, Gemechu Tadesse, Getachew Tollera, Abebe A. Fola, Jeffrey A. Bailey, Jonathan J. Juliano, Jonathan B. Parr, Melkamu Abte, Ashenafi Assefa

**Affiliations:** Ethiopian Public Health Institute; Ethiopian Public Health Institute; Ethiopian Public Health Institute; Ethiopian Public Health Institute; Ethiopian Public Health Institute; Ethiopian Public Health Institute; Ethiopian Public Health Institute; Ethiopian Public Health Institute; Ethiopian Public Health Institute; Ethiopian Public Health Institute; Ethiopian Public Health Institute; Ethiopian Public Health Institute; Ethiopian Public Health Institute; Ethiopian Public Health Institute; Ethiopian Public Health Institute; Ethiopian Public Health Institute; Brown University; Brown University; Institute for Global Health and Infectious Diseases; Institute for Global Health and Infectious Diseases; Ethiopian Public Health Institute; Ethiopian Public Health Institute

**Keywords:** Ethiopia, Malaria, ACT-resistance, ART-R, PfSMARRTer

## Abstract

**Background:**

Ethiopia experienced a marked resurgence of malaria in 2024. Artemisinin-based combination therapies (ACTs) are first-line treatment for uncomplicated *Plasmodium falciparum* malaria and threatened by the emergence of artemisinin partial resistance (ART-R), associated with mutations in the *P. falciparum kelch13* (*k13*) gene, that could undermine treatment efficacy and accelerate transmission.

**Methods and material:**

We used the national Public Health Emergency Management (PHEM) surveillance system to characterize malaria resurgence and further investigate antimalarial drug resistance markers among cases of recurrent clinical malaria in three selected resurgence sites in central Ethiopia.

**Results:**

Parasite isolates from 15 patients with confirmed clinical recurrent *P. falciparum* malaria were genotyped for molecular markers associated with drug resistance, including mutations in *k13, pfcrt*, *pfmdr1, pfdhfr*, and *pfdhps* using PfSMARRTer multiplex amplicon sequencing in Addis Ababa. Clinical presentation and treatment history were reviewed alongside genotyping results. Three patients (3/15, 20%) with confirmed recurrence were infected by parasites carrying the WHO-candidate ART-R molecular marker K13 P441L. Markers of resistance to other antimalarial drugs were largely fixed in the population. These cases occurred in the context of increasing malaria incidence, with evidence of clonal expansion or dominance of a related lineage. The findings indicate the presence of ACT resistance-associated markers within genetically heterogeneous parasite populations.

**Conclusion:**

The current study documents cases of recurrent parasitemia caused by *P. falciparum* with K13 P441L during the malaria resurgence in the Oromia region. The detection of multiple independent resistance markers suggests ongoing drug pressure on first-line treatments. These findings underscore the need for strengthened molecular surveillance integrated with routine case monitoring to inform treatment policy and support malaria control and elimination efforts in Ethiopia.

## Introduction

Historically, Ethiopia has faced past malaria resurgence, including a major surge in cases in 2023–24, when over 10 million cases were reported compared to around 1 million cases in 2019 [1, 2]. The resurgence has led to an uptick in malaria cases and irregular outbreaks in areas once considered malaria-free, including highland regions, as well as an increase in asymptomatic cases [3, 4]. This poses a significant challenge to established malaria control measures and has set back progress made over the past decades [4–6].

Since 2004, artemisinin-based combination therapy (ACT), primarily artemether–lumefantrine (AL), has been the first-line treatment for uncomplicated *P.falciparum* malaria in Ethiopia. Although most therapeutic efficacy studies reported high cure rates [7–9], artemisinin partial resistance (ART-R) has become a major concern [6, 10–14], suggesting the need for further molecular surveillance of antimalarial drug resistance markers [11].

Artemisinin based combination therapy (ACT) combines a fast-acting artemisinin derivative with a longer acting partner drug to ensure rapid parasite clearance and sustained post-treatment suppression. The artemisinin component is rapidly converted to its active metabolite dihydroartemisinin (DHA) and has a short half-life (approximately 1–2 hours), leading to a marked reduction in parasite biomass within the first 48–72 hours. In contrast, the partner drug has a longer elimination half-life, often persisting for days to weeks after treatment completion. During this elimination phase, parasites may be exposed to declining, subtherapeutic drug concentrations, creating a selective window that favors parasites with reduced susceptibility to the partner drug. Consequently, recurrent parasitemia occurring within the first few weeks after ACT is more consistent with recrudescence or reinfection involving parasites with decreased partner-drug sensitivity, particularly in moderate- to high-transmission settings[15].

In response to the malaria resurgence, this study investigated patients who were returning after previous treatment for malaria to the Gelana Primary Hospital and Tore Health Center between August 10 and September 17, 2024, in West Guji, Gelana Woreda, Oromia regional state, Ethiopia. We enrolled Patients who were seeking treatment for malaria-like illness within 6 weeks of treatment [16]. First, we confirmed the resurgence of malaria in this region using national surveillance data from 2019 to 2024. Samples from enrolled participants were then screened by real-time PCR and samples positive for *Plasmodium falciparum* underwent amplicon deep sequencing using *PfSMARRTer* to characterize antimalarial resistance polymorphisms and parasite diversity. The resulting molecular resistance pro les were linked with patient clinical and treatment data to explore the relationship between molecular resistance markers and clinical outcomes.

## Methods

### Malaria case data 2019–2024

The Ethiopian Public Health Institute (EPHI) has surveillance infrastructure, which allows it to obtain epidemiologic data from sources using both an active surveillance and a passive surveillance (weekly report) system. The surveillance unit of the Public Health Emergency Management (PHEM) department at EPHI collects data on epidemic-prone diseases from the health facility level, which is aggregated by catchment, district, zone, and region. Weekly clinical malaria surveillance data used in this study were obtained from the PHEM surveillance unit spanning January 2019 to December 2024.

### Study population, data collection and analysis

Patients who re-visited the selected health facilities after prior treatment with suspected malaria and who had a positive blood smear within 42 days after their initial treatment during the study period were enrolled.

Demographic, clinical, and laboratory test data were collected from all consented participants by trained health workers at the Outpatient Department (OPD) and/or laboratory in each study health facility. Eligible patients’ medical record numbers (MRN) were identified. After routine malaria diagnosis by blood lm an additional 2–3 ml of venous blood, stored at 2–8 °C and transported to the EPHI laboratory for further investigation.

A structured questionnaire was used for data collection including demographic characteristics, malaria diagnosis and treatment history, additional data from the registries were collected using MRN. Data collectors were trained using a pre-tested questionnaire to ensure data quality.

Real-time PCR [17]was used to determine species of infection. In brief, pan-*Plasmodium* specific primers and probe sets targeting the small subunit ribosomal RNA (18S rRNA) along with primers and probes specific for the var gene acidic terminal sequence (ATS) of *P. falciparum*. The samples were further investigated with multiplexed primers for *P. falciparum* (varATS) and Plasmodium *vivax*’s small subunit ribosomal RNA (P.v18S rRNA) (Belachew et al, 2021). DNA samples con rmed with *P. falciparum* mono-infection and with a cycle threshold (Ct) value below 30 were further sequenced using PFSMARRTer V13 [18] on the NextSeq 550 Illumina platform at EPHI.

### Data analysis

Data were analyzed using the R software (v4.5.1). Descriptive analysis was done to describe the study variables and outcomes. Trend analysis specific to the Oromia Region was conducted from the weekly epidemiological malaria data from 2019 to 2024. The mean plus two standard deviations of the baseline data was analyzed to calculate the statistical threshold for each epidemiological week. The diagnostic performance of the blood lm was determined for identification of *P. falciparum* and *P. vivax* by considering real time PCR as the reference method. The Cohen’s Kappa coefficient was computed to measure agreement between microscopy and real time PCR classifications [19].

Sequencing data was processed by SeekDeep v3.0.1 (https://github.com/bailey-lab/SeekDeep). The prevalence of molecular markers associated with antimalarial drug resistance was calculated as the proportion of successfully sequenced infections harboring the mutant allele among the total number of genotyped samples. The R v4.5.2, *tidyverse, ggplot2* package was used for visualization. Complexity of infection (COI) was estimated using sample allele frequency data across polymorphic loci included in the sequencing panel [20]. Genetic relatedness among parasite isolates was estimated using the *hmmIBD* method[21]. Parasite relatedness networks were constructed and visualized using the *igraph* package in R.

### Ethical clearance

This study obtained approval from the scientific and review committee of the EPHI. Permission was obtained from EPHI to use the national EPI-week-based malaria data. Data were collected from consented patients as per the Helsinki principles.

## Results

### Malaria epidemiology in Oromia Region

**Figure 1:**
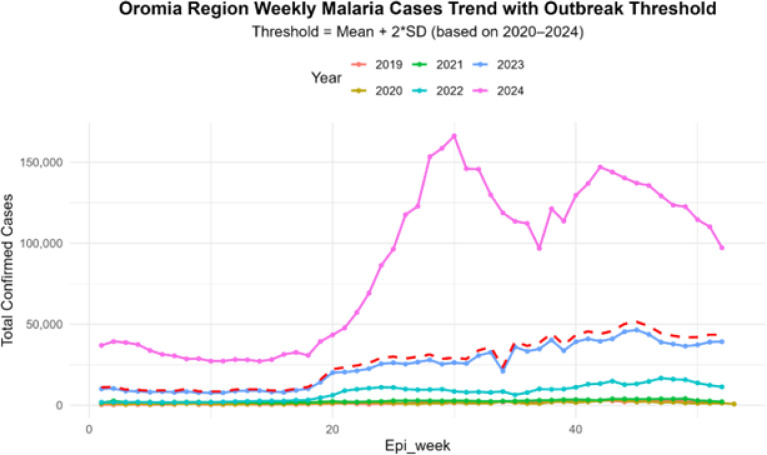
Weekly confirmed malaria case trend during 2019 to 2024 in Oromia Regional State, Ethiopia. The weekly case counts for the region are shown by each colored line, with the resurgence year (2024) in pink. The dashed red line represents the outbreak threshold, defined as a five-year weekly baseline mean plus two times standard deviation.

Analysis of PHEM weekly surveillance data from 2019–2024 demonstrates a steadily increasing malaria burden, with reported cases rising from 75,854 in 2019 to 4,489,228 in 2024 (Fig. 1). Analysis of trends in 2024 highlight a sustained resurgence throughout 2024, during which malaria incidence exceeded the outbreak threshold in every epidemiologic week (Fig. 1). The epidemic curve showed two distinct transmission peaks: an initial rise between epidemiologic weeks 20–30, followed by a larger and more prolonged peak between weeks 35–43.

### Socio-demography and clinical characteristics of the study participants

Samples were collected from 34 study participants seeking repeated treatment. Complete clinical and socio-demographic data were available for 31 (92%) (Table 1). A microscopic parasite species identification at health facility showed that a total of 27 patients were diagnosed with *p. falciparum* during the second visit compared with 23 patients during the first visit, of whom 20 were consistently diagnosed with *p. falciparum* at both visits (Table 1).

Among the 31 participants, 18 (58.1%) were male (Table 2). The median age was 25 years (IQR = 20) (Table 2). The mean number of days between the initial treatment and second visit to the health facility was 15.65 (SD = 7.45; range, 6–26) for *P. falciparum* and 15.75 (SD = 7.48; range, 6–27) for *P. vivax* cases. Almost half 17 (54.84%) of the patients visited the health facilities for the second time within the first two weeks after completion of the initial treatment. The majority of the patients 29(93.55%) were treated with artemether-lumefantrine (AL) and single-dose primaquine 27(87.1%) during the second course of the treatment (Table 2). 11(91.67%) patients with *P. Vivax* infection were treated with AL due to misdiagnosis by microscopy.

### Microscopy and real-time PCR result agreement

Microscopy results from the health facility reported 30 (88.2%), 2 (5.9%) and 2 (5.9%) of *P. falciparum, P. vivax*, and mixed species infections, respectively. Out of 34 samples, real-time PCR identi ed *P. falciparum* in 15 (44.1%), *P. vivax* in 13 (38.2%), mixed in 2 (5.9%), and no malaria parasite in 4 (11.8%) samples.

From the 26 samples classified as *P. falciparum* by microscopy, 14 (53.8%) were confirmed as *P. falciparum* by PCR, while the remaining 11 (42.3%) were identified as *P. vivax*. The result from microscopy performance showed 100% (14/14) sensitivity for detecting *P. falciparum*, but much lower specificity at 8.3% (1/2) for *P. falciparum* with PCR as the gold standard. Sensitivity and specificity for *P. vivax* was 8.3% (1/12) and 100% (14/14), respectively. The overall species-level agreement between microscopy and PCR was low, with a Cohen’s Kappa of 0.09 (95% CI: − 0.08 to 0.26).

#### Quality of amplicon sequencing of P. falciparum infections

**Figure 2:**
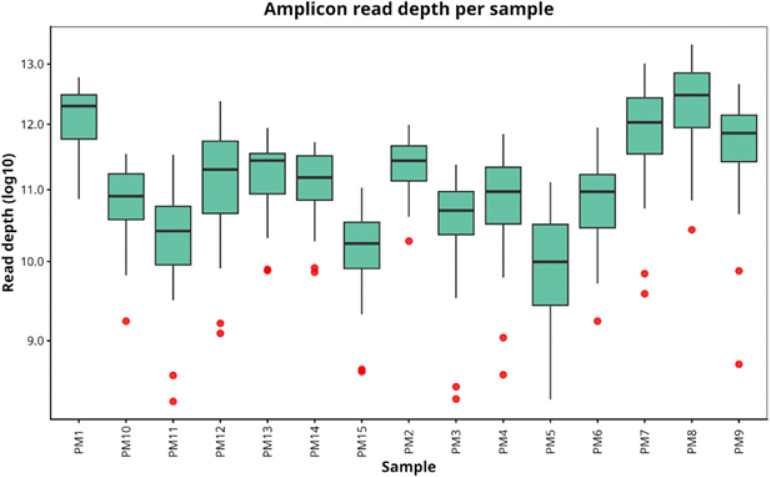
Amplicon read depth distribution per sample among 15 falciparum samples. Box plots of the distribution of read depth across loci in a sample is shown. Read depth is shown on log_10_ scale. Red points indicate outliers.

Amplicon sequencing of 24 *P. falciparum* loci across 15 clinical isolates generated uniformly high coverage. Median read depth per amplicon was 8,124× (interquartile range: 4,210 15,630×), with more than 92% of locus-sample combinations exceeding 1,000× coverage. This depth enabled reliable detection of minor haplotypes at frequencies as low as 1%. Seventeen low coverage outliers, marked in red (Fig. 2), corresponded to samples with low parasitemia, as confirmed by quantitative PCR.

### Cases of participants returning with symptomatic malaria and genetic markers of ART-R

Analysis of samples from 15 participants revealed the presence of the WHO-candidate ART-R marker *k13* P441L mutation in three cases, detailed below.

#### Case 1

A 10-year-old female patient presented to the outpatient department (OPD) with a chief complaint of severe headache and high-grade fever on 25 August 2024. The patient was diagnosed positive for mixed malaria parasite infection (*P. falciparum and P. vivax*) by microscopic investigation of peripheral blood film. The patient was critically ill and subsequently admitted and given initial treatment of intravenous artesunate followed by Artemether/Lumefantrine and a full dose of primaquine for mixed infection in accordance with the national first-line antimalarial treatment guideline. The patient returned to the health facility with recurrent symptoms fourteen days (on 8 September 2024) after the completion of the initial *P. falciparum* treatment course. Repeated blood film microscopy test confirmed *P. falciparum*. A blood sample was collected and sent to the National Reference Laboratory for further molecular investigation.

#### Case 2

A 43-year-old male patient who presented with severe back pain confirmed for *P. falciparum* malaria parasite on 10 August 2024. Following the national malaria treatment protocol, the patient was treated with a full course of artemether-lumefantrine followed by single dose primaquine. Eight days after completion of the initial treatment course, the patient returned to the same health facility on 22 August 2024 with recurrent symptoms, and the microscopic investigation revealed the presence of *P. falciparum*.

#### Case 3

A two-year-old female patient presented with high-grade fever and chills was confirmed for *P. falciparum* after blood lm microscopic examination on 29 July 2024. The patient was treated with a complete course of artemether-lumefantrine followed by single dose primaquine. The patient returned to the same health facility with recurrent symptoms on August 16, 2024, fifteen days after the completion of the initial treatment course. The repeated microscopy investigation detected *P. falciparum*.

### Prevalence of Non-ACT and Partner Drug Resistance Mutations

**Figure 3:**
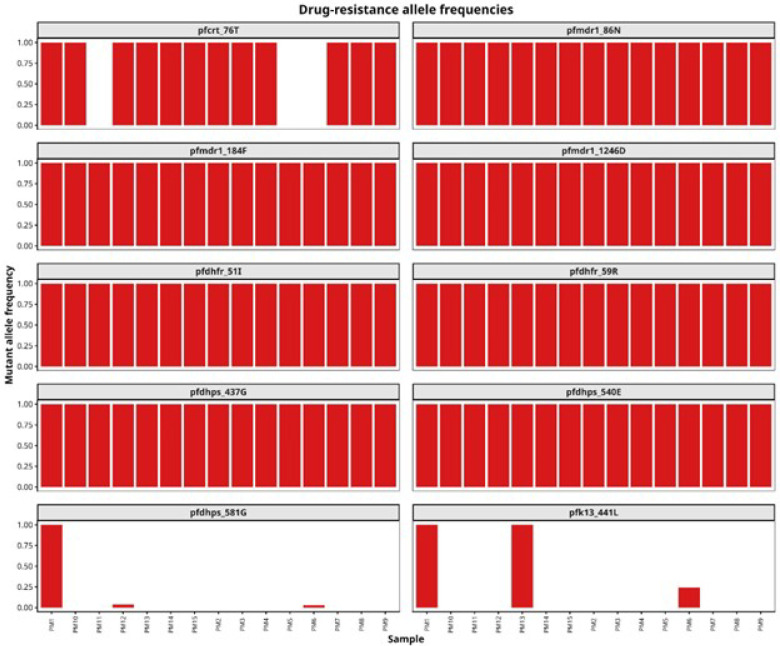
Within-sample drug-resistance mutation frequencies for 15 falciparum infections. Stacked bar plot illustrating mutant (red) and wild type otherwise, *pfcrt* has three missing data points. When detected, all antimalarial resistance polymorphisms occurred at 100% within sample frequency, except *dhps* A581G and *k13* P441L. Three samples fail sequencing for *pfcrt* 76T.

Amplicon sequencing showed that molecular markers for many antimalarials were common in the sequenced isolates including *pfcrt (PfcRT_76T), pfmdr1 (N86, 184F, D1246), pfdhfr (51I, 59R,108N)*, and *pfdhps* (540E). The *pfdhps* 581G and k13 441L were detected in three samples each 3/15 (20%). Within-sample allele frequencies of each mutation are shown in Fig. 3, showing that in most cases, samples contained a single allele at these loci. A sextuple mutant haplotype combining *pfcrt* 76T, the *pfmdr1* NFD triple, the *pfdhfr* IRN triple, and *pfdhps* 437G/540E occurred in almost all the samples, except three missing data for *pfcrt* 76T Fig. 3.

### Complexity of Infection (COI)

**Figure 4:**
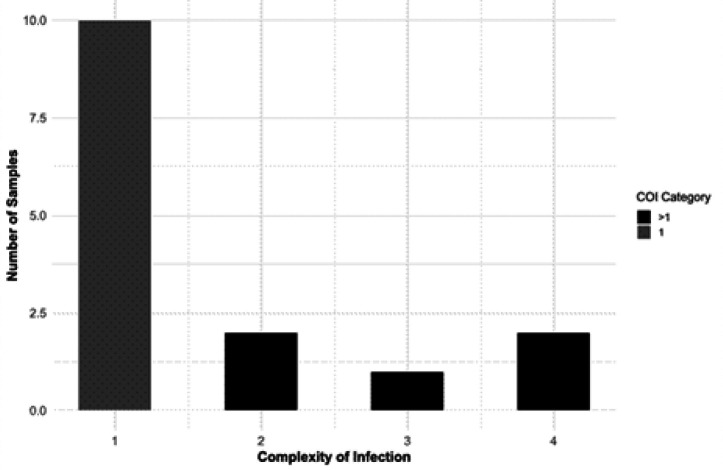
Distribution of the Complexity of Infection (COI) among fifteen P. falciparumsamples.

The majority of *P. falciparum* isolates were monoclonal 10/15(66%). Ten isolates exhibited a COI of 1, while a smaller proportion showed higher COI values, including COI = 2 (n = 2), COI = 3 (n = 1), and COI = 4 (n = 2) (Fig. 4).

### Genetic diversity and relatedness

Haplotype clustering at 99% nucleotide identity revealed pronounced variation in polyclonality (Fig. 4). The ama1 locus displayed the highest diversity, with up to four haplotypes detected in sample PHEM-6 and a mean complexity of infection (COI) of 1.9 ± 0.8 across all samples (Fig. 4). The heome loci cluster exhibited intermediate diversity, with mean COI values ranging from 1.6 to 1.8. In contrast, drug-resistance loci (*pfcrt, pfmdr1, pfdhfr, pfdhps, pfk13*) were monoclonal (COI = 1) in nearly all instances, and 66% of isolates (10 of 15) showed COI = 1 across all resistance markers (Fig. 4). Mean COI was significantly higher in neutral and antigenic loci than in mutated genes (Wilcoxon rank-sum test, P < 0.001).

**Figure 5:**
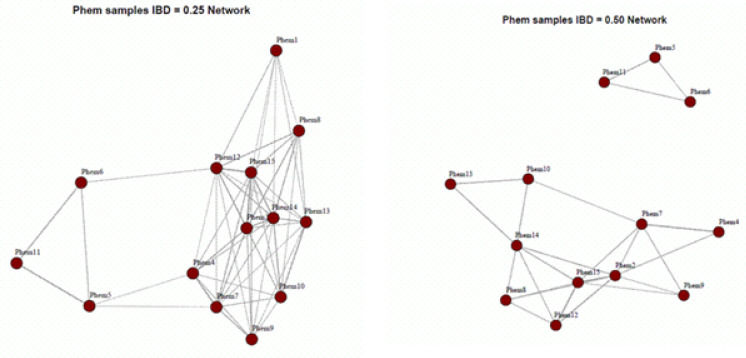
Genetic relatedness among samples based on estimated Identity By Descent (eIBD) analysis. Each dot represents an individual sample, and connecting lines indicate sample pairs with IBD values at or above the defined threshold, reflecting close genetic relatedness. The IBD value denotes the proportion of the genome shared identically by descent between two parasite isolates. Notably, samples PHEM1, PHEM6, and PHEM13 harbor the 441L mutation; however, in PHEM6 the mutation is present at a minor allele fraction of 24.3%, suggesting within-host heterogeneity rather than a fully fixed variant.

Although the sample size is limited, Fig. 5 highlights notable genetic clustering among parasites carrying the 441L mutation. At an IBD threshold of 0.50 (indicative of sibling-level relatedness), 441L mutant parasites form distinct high-relatedness clusters, consistent with recent clonal expansion and focal transmission amplification. At a lower threshold (IBD = 0.25), these mutants remain genetically connected to a broader parasite population, suggesting ongoing gene ow within the study area.

Together, these patterns support the interpretation that the increase in 441L prevalence is likely driven by local expansion of established parasite lineages rather than repeated introductions of genetically divergent strains.

## Discussion

Ethiopia experienced a marked resurgence of malaria during 2024, with Oromia Region contributing substantially to the national increase in cases. In this investigation of recurrent clinical malaria cases from Gelana Woreda, we combined surveillance data, clinical characterization, and targeted amplicon sequencing to examine whether antimalarial drug resistance may be occurring during early recurrence. Our findings demonstrate four key observations: (a) sustained resurgence throughout 2024 in the study region; (b) low agreement between routine microscopy and molecular species identification; (c) the presence of WHO-validated *pfk13* P441L mutations among recurrent *Plasmodium falciparum* infections and (d) there are high levels of molecular markers of antimalarial resistance in these recurrent parasitemias.

The discordance between microscopy and qPCR species identification was substantial, with very low agreement (κ = 0.09). Although microscopy remains the cornerstone of malaria diagnosis in Ethiopia, this level of misclassification—particularly between *P. falciparum* and *P. vivax*—has important implications in a resurgence setting. Species misidentification may lead to suboptimal treatment selection, inappropriate use of primaquine, and inaccurate surveillance data [22, 23]. These findings reinforce the need to strengthen diagnostic quality assurance systems and consider strategic incorporation of molecular confirmation in sentinel or outbreak settings.

Among the 15 qPCR-confirmed *P. falciparum* infections that underwent deep amplicon sequencing, three harbored the WHO-validated *pfk13* P441L mutation [24]. These three cases returned with recurrent parasitemia 8–15 days after completion of artemether–lumefantrine (AL) treatment. Although we cannot definitively distinguish recrudescence from reinfection in this small cohort, the temporal proximity of recurrence and the presence of validated artemisinin partial resistance (ART-R) markers are notable. These findings align with recent reports documenting geographically heterogeneous emergence of *pfk13* mutations in Ethiopia, including R622I, P574L, A675V, and P441L [9, 11, 12]. The P441L mutation has been reported from geographical proximate areas in low prevalence 1–2% in Oromia and Central Ethiopia regions [9, 11]. A recent work on malaria resurgence in Ethiopia reported 441L mutations in 9 of 22 samples in Dila, Central Ethiopia (In press). Given the limited number of samples, IBD sharing supports the previous reports that malaria resurgence in the area may not be independent, implicating a sustained transmission of similar lineage may be due expansion of pre-existing parasite lineages[9].

In addition to artemisinin resistance markers, we observed near-fixation of mutations in *pfcrt, pfmdr1, pfdhfr*, and *pfdhps*. The dominance of the sextuple mutant haplotype (including *pfcrt 76T, pfmdr1* NFD, *pfdhfr* IRN, and *pfdhps* 437G/540E) reflects longstanding antifolate and chloroquine selection pressure in Ethiopia [11, 12]. Of particular relevance is the high prevalence of the *pfmdr1* N86 allele, previously associated with altered lumefantrine tolerance, which was present at 100% frequency within all parasitemias genotyped.

Fortunately, ACT therapeutic efficacy studies in Ethiopia have continued to report PCR-corrected cure rates above 95%. However, the molecular landscape suggests important changes that need continued monitoring [12]. Continued reliance on AL as first-line therapy for *P. falciparum*, particularly during periods of intense transmission, may further select for parasites with reduced partner-drug susceptibility, potentially accelerating the clinical impact of emerging artemisinin resistance, as has been seen with the emergence of the PX1 PIN haplotype in Uganda [25].

This study provides, to our knowledge, the first molecular documentation of artemisinin partial resistance markers among clinically recurrent malaria cases in Ethiopia. Malaria incidence reportedly declined in 2025, suggesting that resurgence dynamics are likely multifactorial and not solely attributable to drug resistance. However, the detection of multiple independent ART-R markers, both here and in other studies [9, 11, 12], in a resurgence setting signals ongoing evolutionary pressure and underscores the need for strengthened surveillance.

Several limitations should be considered. The sample size was small, and sites were purposefully selected based on recurrent presentations, limiting generalizability. We did not have initial parasitemia to genotype. We did not perform whole genome sequencing or pharmacokinetic analyses to definitively distinguish recrudescence from reinfection or to assess drug exposure. In addition, diagnostic discrepancies between microscopy and qPCR may have influenced case classification. Nonetheless, the integration of epidemiologic, clinical, and high-depth amplicon sequencing data provides a coherent signal that resistance-associated polymorphisms are present among recurrent cases in this resurgence context.

In this case series from a district experiencing malaria resurgence, we identified circulating ART-R–associated mutations within genetically related *Plasmodium falciparum* parasite populations, in an area with the absence of clear evidence of artemisinin-based combination therapy (ACT) clinical failure. Although standard treatment outcomes remain largely preserved at the population level, the genomic findings raise concern for emerging resistance within the region. The findings of this work underscore the importance of integrating molecular surveillance with routine case detection, strengthening diagnostic and laboratory capacity, and conducting timely therapeutic efficacy studies to contextualize genomic signals. Proactive genomic monitoring during periods of resurgence may enable malaria control programs to identify early changes in parasite susceptibility and adjust treatment policy before widespread clinical failure becomes apparent.

## Supplementary Material

This is a list of supplementary les associated with this preprint. Click to download.

• Annex.docx

## Figures and Tables

**Table 1:** Results of microscopic identification of parasite species during the subsequent visit at two health facilities in Gelana Woreda, Oromia Region, Ethiopia (n = 31).

Initial Diagnosis	Second visit Diagnosis			
	Mixed	Pf.	Pv.	Total
Pf.	2 (8.7%)	20 (87.0%)	1 (4.3%)	23
Pv.	0	6 (85.71%)	1 (14.29%)	7
Mixed	0	1 (100%)	0	1
Total	2	27	2	31

**Table 2:** Socio-demographic and clinical profile of malaria patients confirmed by PCR from Gelana Woreda, Oromia Region, Ethiopia (n = 31).

Variables	Category	Pf. (n = 14)	Pv. (n = 12)	Neg (n = 3)	Mix (n = 2)	Total
Sex	Male	7 (50%)	8(66.67%)	2(66.67%)	1(50%)	18(58.1%)
	Female	7 (50%)	4(33.3%)	1(33.33%)	1(50%)	13(41.94%)
	**Total**	**14**	**12**	**3**	**2**	**31**
Duration between initial and second visit	< 14	8(57.14%)	7(58.33%)	2(66.67%)	0	17(54.84%)
	14–21	1(7.14%)	0(0%)	1(33.33%)	1(50%)	3(9.68%)
	21 and above	5(35.71%)	5(41.67%)	0	1(50%)	11(35.48%)
	**Total**	**14**	**12**	**3**	**2**	**31**
Treatment given on second visit	AL	14(100%)	11(91.67%)	3(100%)	1(50%)	29(93.55%)
	No AL	0	1(8.33%)	0	1(50%)	2(6.45%)
	**Total**	**14**	**12**	**3**	**2**	**31**
	Full dose Primaquine	1(7.14%)	2(16.67%)	0	1(50%)	4(12.9%)
	Single dose Primaquine	13(92.86%)	10(83.33%)	3(100%)	1(50%)	27(87.1%)
	**Total**	**14**	**12**	**3**	**2**	**31**
First symptom	Back pain	0	0	1(33.33%)	0	1(3.23%)
	Chills and rigor	2(14.29%)	3(25%)	0	0	5(16.13%)
	Fever	8(57.14%)	5(41.67%)	0	0	13(41.94%)
	Headache	4(28.57%)	3(25%)	2(66.67%)	1(50%)	10(32.26%)
	Shivering	0	1(8.33%)	0	1(50%)	2(6.45%)
	**Total**	**14**	**12**	**3**	**2**	**31** *

Pf.= *P. falciparum*, Pv. =*P. vivax*, AL = artemether-lumefantrine

* Two cases, one *P. falciparum* and one *P. vivax*, were excluded from further analysis due to incomplete clinical and demographic profile.

**Table 3:** Microscopy and real-time PCR test results of the microscopy-confirmed *P. falciparum* samples collected from Gelana Woreda, Oromia Region, Ethiopia (n = 26)

Result		real time PCR		Total
		Pf.	Pv.	
**Microscopy**	Pf.	14	11	25
	Pv.	0	1	1
**Total**		14	12	26*

Pf.= *P. falciparum*, Pv. =*P. Vivax*

* Two real-time positive cases, one *P. falciparum* and one *P. vivax*, were excluded from further analysis due to incomplete clinical and demographic profile.

## Data Availability

Data is available from the corresponding author with a modest request.

## Abbreviations

CT: Artemisinin-based Combination Therapy
AL: Artemether Lumefantrine
ART-R: Artemisinin Partial Resistance
K13: Kelch 13 propeller domain
pfk13: P. falciparum kelch 13 gene
*pfcrt*: *P. falciparum* chloroquine resistance transporter
*pfmdr1*: *P. falciparum* multidrug resistance 1
*pfdhfr*: *P. falciparum* dihydrofolate reductase
*pfdhps*: *P. falciparum* dihydropteroate synthase
rPCR: realtimePolymerase Chain Reaction
PfSMARRTer: *Plasmodium falciparum* Streamlined Multiplex Antimalarial Resistance and Relatedness
NGS: Next-Generation Sequencing
SNP: Single Nucleotide Polymorphism
MOI: Multiplicity of Infectio
COI: Complexity of Infection
PHEM: Public health emergency management
EPHI: Ethiopian public Health institute
